# Primary anastomosis as a valid alternative for extremely low birth weight infants with spontaneous intestinal perforation

**DOI:** 10.1007/s00431-021-03926-2

**Published:** 2021-01-12

**Authors:** Martin Dübbers, Gerd Holtkamp, Grigore Cernaianu, Marc Bludau, Janina Fischer, Titus Keller, Angela Kribs, Daisy Schulten

**Affiliations:** 1 Division of Pediatric Surgery, Medical Faculty and University Hospital Cologne, University of Cologne, Kerpener Strasse 62, 50937 Cologne, Germany; 2 Department of General, Visceral and Cancer Surgery, Medical Faculty and University Hospital Cologne, University of Cologne, Cologne, Germany; 3 Department of Pediatrics, Medical Faculty and University Hospital Cologne, University of Cologne, Cologne, Germany

**Keywords:** Extremely low birth weight, ELBW, Focal intestinal perforation, Primary anastomosis, Spontaneous intestinal perforation

## Abstract

The aim was to assess the results of primary anastomosis (PA) compared to enterostomy (ES) in infants with spontaneous intestinal perforation (SIP) and a weight below 1000 g. Between 2014 and 2016, enterostomy was routinely carried out on extremely low birth weight (ELBW) patients with SIP. From 2016 until 2019, all patients underwent anastomosis without stoma formation. We compared outcome and complications in both groups. Forty-two patients with a median gestational age of 24.3 weeks and a birth weight of 640 g with SIP were included. Thirty patients underwent PA; ES was performed in 12 patients. Overall in-hospital mortality was 11.9% (PA: 13.3%, ES: 8.3%). Reoperations due to complications became necessary in 10/30 patients with PA and 4/12 patients with ES. Length of stay was 110.5 days in the PA group and 124 days in the ES group. Median weight at discharge was higher in the PA group (PA: 2258 g, ES: 1880 g, *p* = .036).

*Conclusion*: Primary anastomosis is a feasible treatment option for SIP in infants < 1000 g and may have a positive impact on weight gain and length of hospitalization. However, further studies on selection criteria for PA are necessary.

**Table:** 

**What is Known:** *• Enterostomy (ES) and primary anastomosis (PA) are feasible treatment options in preterm infants with spontaneous intestinal perforation (SIP).* *• Stomal complications or failure to thrive due to poor food utilization can pose significant problems.*	
**What is New:** *• Primary anastomosis in case of SIP is equal to enterostomy in terms of mortality and revision rate; however, length of stay and weight gain can be presumably positively influenced.* *• Primary anastomosis is a valid treatment option even for patients weighing less than 1000 g.*

**What is Known:**

*• Enterostomy (ES) and primary anastomosis (PA) are feasible treatment options in preterm infants with spontaneous intestinal perforation (SIP).*

*• Stomal complications or failure to thrive due to poor food utilization can pose significant problems.*

**What is New:**

*• Primary anastomosis in case of SIP is equal to enterostomy in terms of mortality and revision rate; however, length of stay and weight gain can be presumably positively influenced.*

*• Primary anastomosis is a valid treatment option even for patients weighing less than 1000 g.*

## Introduction

The great advances in neonatology led to a significant reduction in premature infant mortality. At the same time, however, the surgeon is now confronted with smaller neonates requiring surgical therapy. Spontaneous intestinal perforation (SIP) is a typical complication in extremely immature infants sometimes weighing significantly less than 1000 g. The etiology of this lesion has not been unequivocally clarified yet. However, at least in terms of therapy and outcome, it must be differentiated from necrotizing enterocolitis (NEC) that is significantly less often etiological in children under 1000 g in their first phase of life.

The type of surgery in case of intestinal perforation has been a matter of debate. Enterostomy (ES) is one of the most common surgical procedures in premature infants with intestinal perforation and probably still the gold standard for extremely low birth weight infants [1]. Complications after enterostomy, such as loss of electrolytes, stoma prolapse, or skin macerations, are common, especially in very young children [2, 3]. Most of all, a failure to thrive due to poor food utilization after enterostomy can pose significant problems for neonatologists. In addition, at least one more surgery under general anesthesia will be necessary in the further course.

Therefore, avoiding an enterostomy in case of intestinal perforation seems to be generally advantageous. In the literature, there are retrospective single-center examinations on the results of enterostomy compared to primary anastomosis (PA) in premature infants with intestinal perforation [1, 4, 5]. However, especially for the group of premature infants weighing less than 1000 g at surgery, data of larger patient cohorts are missing.

In our institution, primary enterostomy was performed in patients with spontaneous intestinal perforation weighing less than 1000 g from November 2014 to June 2016. Subsequently, until October 2019, primary anastomosis was performed as a first surgery of these children. We evaluated and compared both treatment methods regarding outcome, complication rate, mortality, and incidence of required re-interventions.

## Methods

### Study cohort

**Fig. 1:**
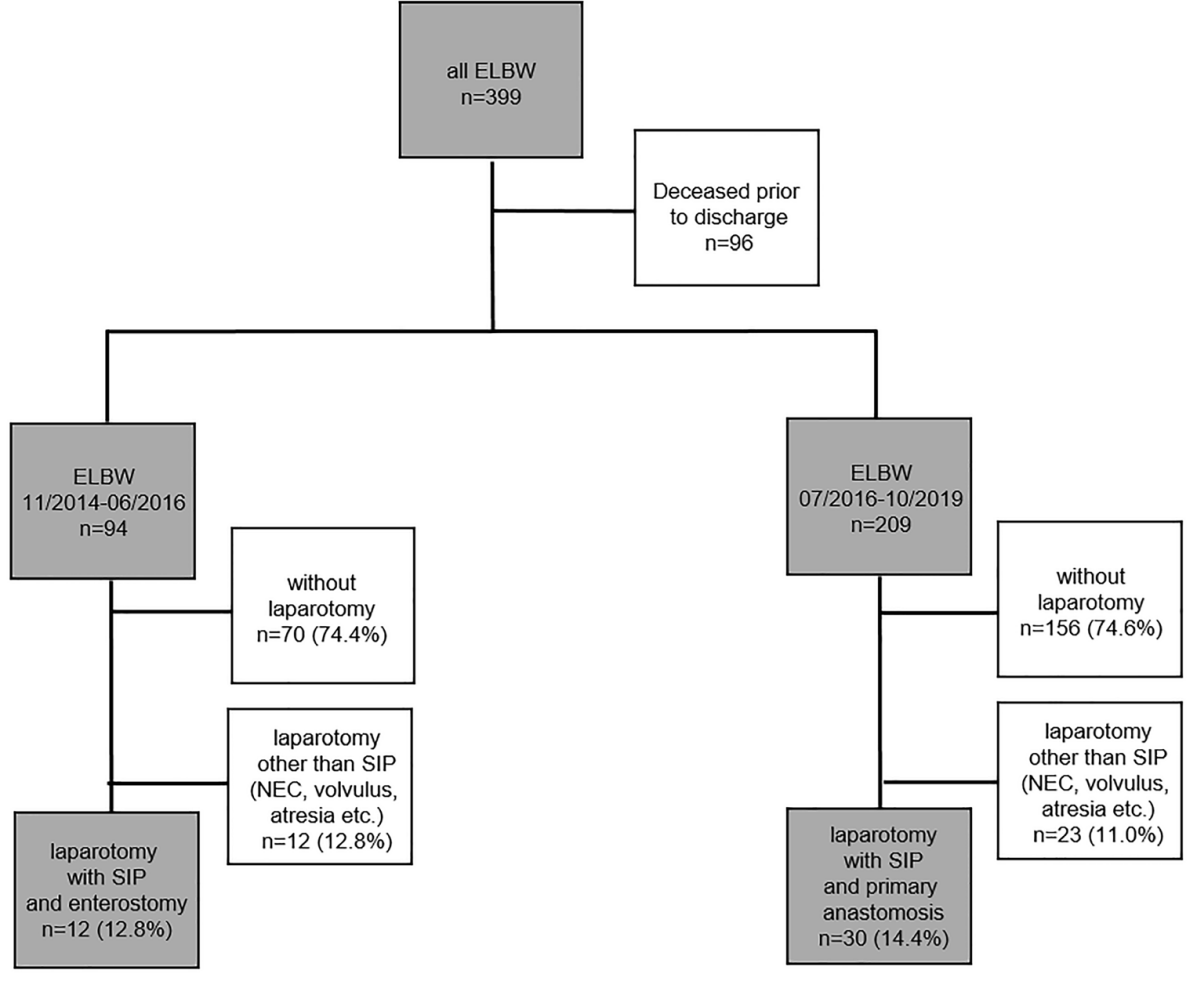
Flow chart of all patients with extremely low birth weight (ELBW) in our study period from June 2016–Oct 2019. SIP, spontaneous intestinal perforation; NEC, necrotizing enterocolitis

The data of all extremely low birth weight (ELBW) infants weighing less than 1000 g that underwent a laparotomy due to intestinal perforation between November 2014 and October 2019 were analyzed retrospectively. Between November 2014 and June 2016, these children routinely underwent enterostomy with or without bowel resection in our institution. In the subsequent period from August 2016 to October 2019, primary anastomosis without stoma formation was performed. Patients showing signs of necrotizing enterocolitis and gastric perforations or patients that underwent an exploratory laparotomy regardless of any surgical technique were excluded from the study (Fig. 1). In addition to gender, age, gestational age, and birth weight, the days of life, the weight at surgery, and the duration of surgery were recorded. In terms of outcome, the frequency and cause of reoperations, in-hospital mortality, and length of hospitalization, as well as weight and head circumference at discharge, were examined in both groups. Furthermore, the “Clinical Risk Index for Babies” (CRIB score) at birth was determined as a general preoperative risk assessment. This index considers birth weight, gestational age, congenital malformations, the maximum base excess, and the required maximum inspiratory O_2_ concentration in the first 12 h of life [6].

### Statistical analysis

The univariate statistical analysis of the data was carried out using Fisher’s exact test for categorical variables and the Mann-Whitney test for non-categorical variables. The analysis was carried out by means of the SPSS® 25 software (IBM®, Munich, Germany). A statistically significant difference was assumed at *p* < .05. Survival curves were estimated according to the method of Kaplan and Meier and compared by the log-rank test. Event-free survival (EFS) time was calculated as the time from laparotomy to event or discharge if the patient had no event. Any laparotomy, enterostomy, or wound debridement and death from other diseases or surgery-related were regarded as events. Overall survival (OS) was calculated as the time from laparotomy to death from other diseases or surgery-related or discharge if the patient survived.

## Results

### Patients’ characteristics

Forty-two patients (11 female) met the inclusion criteria. The median gestational age of the patients was 24.3 weeks (22–29.4) and the median birth weight 640 g (260–990). In the first period, laparotomy was performed in a total of 12 patients due to spontaneous intestinal perforation. Enterostomy (ileostomy *n* = 11, jejunostomy *n* = 1) was performed in all patients. Thirty patients underwent surgery in the second period, when primary anastomosis of the intestine was performed without stoma formation. A total of 5/12 patients of the enterostomy group and 23/30 of the primary anastomosis group received a drain before undergoing laparotomy. The patient groups did not differ in gestational age, birth weight, head circumference, age at surgery, and CRIB score. The median age of the patients at the time of surgery was 6 days (2–22). There were no significant differences between the two groups in length of resected bowel and duration of surgery (ES 69 min vs. PA 66 min). The detailed list of the surgery-related data is shown in Table 1.

**Table 1:** Characteristics and outcome of ELBW infants with SIP

	Total cases (*n* = 42)	Enterostomy (*n* = 12)	Anastomosis (*n* = 30)	*p* value
Gestation weeks (median, range)	24.29 (22–29.43)	24.43 (22–29.43)	24.21 (22–27.43)	.486
Birth weight (g) (median, range)	640 (260–990)	640 (260–980)	635 (290–990)	1.000
Head circumference (cm) (median, range)	22 (16.5–26.0)	21.85 (16.5–26.0)	22 (18.0–26.0)	.595
Gender M:F	2.8:1	3:1	2.8:1	1.000
CRIB score (median, range)	9 (1–17)	9 (1–17)	9 (1–16)	.804
Age at operation (days) (median, range)	6 (2–22)	7 (2–20)	5.5 (3–22)	.961
Weight at operation (g) (median, range)	604 (270–980)	625 (270–980)	604 (290–950)	.842
Operation time (min) (median, range)	66 (30–112)	68.5 (30–87)	66 (35–112)	.587
Length of resected bowel (cm) (median, range)	2.0 (0–6)	1.0 (0–4)	2.0 (0–6)	.203
In-hospital mortality	5/42 (11.9%)	1/12 (8.3%)	4/30 (13.3%)	1.000
Survivors	*n* = 37	*n* = 11	*n* = 26	
Length of stay (days) (median, range)	115 (73–267)	124 (73–267)	110.5 (79–173)	.445
Additional length of stay for stoma closure (days) (median, range)		*n* = 6 13 (8–31)		
Weight at discharge (g) (median, range)	2170 (1406–3825)	1880 (1406–3825)	2258 (1800–2980)	.036
Head circumference at discharge (median, range)	32 (29–38)	31.6 (29–34)	32.0 (30–38)	.967
ICH > II°, at discharge (*n*)	11/37 (29.7%)	3/11 (27.3%)	8/26 (30.8%)	1.000

### Surgical revisions

**Fig. 2:**
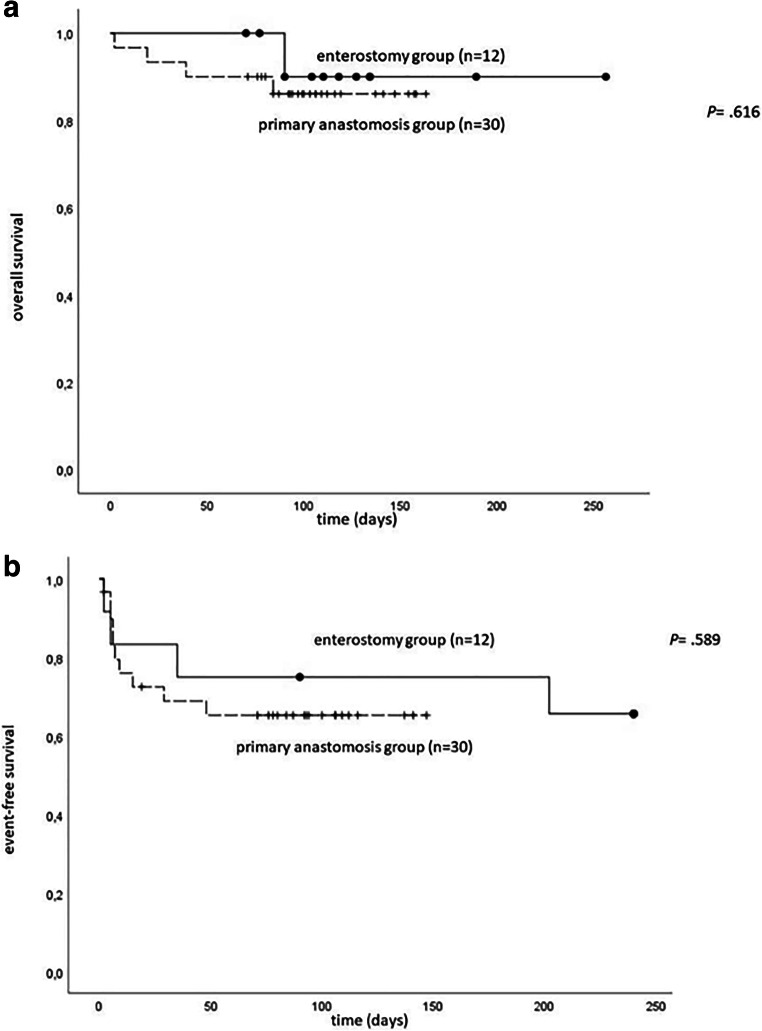
Kaplan-Meier plots of all patients with extremely low birth weight (ELBW) in our study period from June 2016 to Oct 2019. **a** Overall survival of ELBW in the enterostomy group (ES: black line) and in the primary anastomosis group (PA: broken line). **b** Intervention-free survival of ELBW in the enterostomy group (ES: black line) and in the primary anastomosis group (PA: broken line)

In 10/30 patients (33%) of the PA group and 4/12 patients of the ES group (33%), further surgical interventions due to complications were necessary (Table 2). In the PA group, a reoperation was indicated in 6 cases due to reappearing SIP and in one case each due to NEC, adhesive ileus, and wound dehiscence after laparotomy. Anastomotic leak occurred in 1 patient (3.3%). An ileostomy was performed in 3 of these patients as part of the revision. In the ES group, further surgical interventions were necessary due to SIP (*n* = 2) and ileus (*n* = 2). In 2 cases, ileostomy had to be performed again. The two groups did not differ significantly in their event-free survival (Fig. 2b).

**Table 2:** Characteristics of patients with postoperative complications requiring re-intervention (*n* = 14)

	SIP with enterostomy (*n* = 4/12)	SIP with anastomosis (*n* = 10/30)	*p* value
Gestation weeks (median, range)	24.7 (22–29.4)	23.1 (22.6–27.4)	.562
Birth weight (g) (median, range)	775 (430–980)	542 (290–900)	.350
CRIB score (median, range)	6.5 (1–17)	13 (1–16)	.478
Number of re-interventions (*n*) (median, range)	1 (1–3)	1.5 (1–3)	.832
Indication for 1st re-intervention			.832
Insufficiency of anastomosis	0	1	
Ileus	2	1	
De novo bowel perforation	2	6	
NEC	0	1	
Abdominal wound dehiscence	0	1	
Survivors	4/4	8/10	1.000

### In-hospital mortality

In our entire patient cohort, the in-hospital mortality was 11.9% (5/42). In detail, 4/30 patients (13.3%) of the PA group and 1 patient (8.3%) of the ES group died. Overall survival did not differ between PA and ES groups (Fig. 2a). Apparently, the deceased children with a median gestational age of 22.85 weeks (22–24.43) and a median birth weight of 480 g (260–620) were significantly less mature compared to the entire patient cohort (*p* = .011 and *p* = .005, respectively). The median CRIB score of 13 (12–16) was four points higher than the score of the entire patient cohort (*p* = .011). The data of the five deceased patients are summarized in Table 3. One patient died on the second day after surgery due to a hemorrhagic shock with massive cerebral hemorrhage. On the 83rd day after the first surgery, another patient, who was in a state of well-being, fell ill with a foudroyant NEC. Despite prompt surgical re-intervention and maximum sepsis therapy, the child died the following day. The third patient with a birth weight of 290 g died on the 39th day after first intervention due to a CMV infection with consecutive CMV peritonitis leading to a sepsis with lethal outcome that could neither be treated conservatively nor surgically. The last patient of this group died 19 days after surgery due to a protracted shock of unknown origin with acute kidney failure. The deceased patient with ES developed recurrent pulmonary hypertensive crises in existing bronchopulmonary dysplasia and consequently died due to right-sided heart failure 90 days after surgery.

**Table 3:** Characteristics of deceased patients (*n* = 5)

Operation procedure	Gestation (weeks)	Birth weight (g)	Gender (m/f)	CRIB score	Day of death after 1st operation
Enterostomy	24.43	260	f	13	90
Primary anastomosis	22.57	490	f	13	84
Primary anastomosis	22.86	290	m	16	39
Primary anastomosis	22.0	480	m	12	2
Primary anastomosis	22.86	620	m	12	19

### Length of stay

The patients were discharged from hospital under the conditions of cardiorespiratory stability (no apneas or bradycardia), independent drinking of a sufficient amount, weight gain, body temperature stability, and adequate parental competence in caring for the child. The median length of stay of the surviving patients of the PA group was 110.5 days (79–173) in our patient cohort. The in-hospital treatment of the surviving patients of the ES group was 124 days (73–267) (*p* = .445). In 6 of these patients, stoma reversal was already carried out as part of this first inpatient treatment. In 6 patients, this was done during a second inpatient stay. Taking these additional inpatient days into account, the median inpatient stay of the surviving patients in the ES group was 139 days (88–267). In relation to the comparison group, this difference was not statistically significant (*p* = .252).

### Weight and head circumference at discharge

The median weight at discharge was significantly higher in the children with PA than in patients with ES (2257.5 g vs. 1880 g, *p* = .036). Head circumference and frequency of ICH > 2° did not differ in the two groups (PA: 32.0 cm; 30.8% vs. ES: 31.6 cm; 27.3%).

## Discussion

The surgical treatment of very small premature infants with acute abdomen often poses a major challenge for the surgeon. The question of the ideal procedure for intestinal interventions due to focal perforations, necrotizing enterocolitis, or ileus is not uniformly assessed in the literature, and especially the need for a simultaneous stoma formation during a laparotomy is still discussed [3]. The supposed advantages of an intestinal stoma by protecting distal intestinal sections, avoiding suture insufficiencies, or through a possibly faster progression to a normal diet are offset by some disadvantages such as poor food utilization, electrolyte loss, or the risk of complications due to the stoma. These are prolapse, retraction, or skin macerations that can frequently be observed in premature infants [3]. In addition, surgical interventions under general anesthesia in premature infants bear an increased risk of complications and may have a negative impact on the neurological development of these children [7–9]. Therefore, it must be the aim to largely avoid all surgical interventions in this phase of life. For this reason, the placement of a percutaneous peritoneal drainage (PD) under local anesthesia may be performed as the primary therapy in case of intestinal perforation or NEC [10, 11]. With regard to the need for an enterostomy in the context of intestinal perforations in premature infants, only a few comparative studies with larger numbers of patients have been published so far. Singh et al. did not show any significant differences regarding the complication rate and mortality in newborns with FIP and NEC when treated with anastomosis or enterostomy in 2006. In contrast, de Haro Jorge et al. found a higher risk of life-threatening complications in the case of primary anastomosis without stoma in an analysis that solely considered premature infants with focal intestinal perforation [1, 12]. So far, only a few data exist for the special group of ELBW infants weighing less than 1000 g. Brisighelli et al. published their results regarding 40 children weighing less than 1500 g (“very low birth weight”) in 2018 and did not find any significant disadvantages of primary anastomosis in case of focal intestinal perforation for this group [4].

Most of the studies published to date, however, are biased by the individual decision of the surgeon on the chosen surgical procedure, so that despite homogeneous patient cohorts, the comparison of different therapeutic concepts seems to remain difficult.

In our setting, it was possible to exclude this bias and to clearly delimit the patient groups due to the defined periods of the respective treatment methods. With a median patient weight of 640 g and a gestational age of 24.3 weeks, the patient cohort of our study differs significantly from the data previously published and also focuses on surgical interventions that were carried out on patients with a weight of less than 1000 g. In the literature, the general mortality rate of ELBW infants is stated to be from 20 to 50% depending on the author, although it differs considerably even within the group, depending on weight and gestational age [13–15]. Therefore, we also determined the CRIB score to classify the general mortality risk of premature infants in order to further validate our results in both patient groups [6, 16]. There were no significant differences regarding weight, gestational age, and score between the two groups of our patient cohort. Eicher et al. found a mortality rate after surgery of 18% in their series of 28 surgically treated ELBW infants (19 stomata, 6 PD, 1 primary anastomosis, 2 explorative laparotomies) [5]. In our study, the mortality rate of the surgically treated children was 11.9%, regardless of the surgical procedure. This rate is low considering the published general mortality rate of ELBW children. The direct comparison of both surgical procedures showed an in-hospital mortality of 13.3% in the PA group and 8.3% in the ES group. Although this difference is not statistically significant, this tendency may impact further studies on the indications for an enterostomy.

In the case-by-case analysis among the 5 deceased patients, 4 patients had a gestational age of less than 23 weeks. The mortality rate of these extremely immature children is generally reported to be over 80% [17]. The number of necessary reoperations did not differ in the two groups (PA group: 10/30 vs. ES group: 4/12), although it must be taken into account that all patients of the ES group needed further surgical intervention in order to reverse the stoma. In both groups, the most frequent cause for surgical revision was reappearing focal perforation. However, no difference regarding the incidence between the two groups could be determined. Therefore, no advantage of an enterostomy could be demonstrated. An anastomotic leak occurred in only 1 of 30 patients. Thus, primary anastomosis in premature infants is possible due to the high self-healing power of the fetal tissue, regardless of the extent of bacterial contamination and even with the most severe inflammatory changes [18–20].

In our study, the type of surgical procedure showed no significant influence on the length of hospitalization, although the patients with primary anastomosis tended to be discharged earlier. In 6 patients, the planned stoma reversal was carried out only after temporary discharge as part of a second inpatient stay requiring another inpatient stay of 13 days on average. In addition, the weight at discharge was significantly higher when primary anastomosis was carried out. This can be evaluated as another positive aspect of this procedure.

Our study was limited by a few points. So far, for example, there are no valid scores to assess the extent of bacterial contamination or peritonitis. The extent of clinical deterioration and the cardiopulmonary status at the time of the intervention could not be analyzed or correlated. By using the CRIB score, at least an attempt was made to take this into account. Patients with meconium ileus or signs of an NEC were also excluded from this study, although own experiences show that primary anastomosis is often possible in these cases either.

In summary, primary intestinal anastomosis in case of spontaneous intestinal perforation is equal to an enterostomy in terms of mortality and revision rate, even in children with a weight of less than 1000 g. Thus, stoma-associated complications and the need for a planned second surgical intervention can be avoided while weight gain can be presumably positively influenced. Future studies are needed to evaluate whether preoperative parameters, including the CRIB score, could be used as selection criteria for PA.

## Data Availability

Raw data are available upon reasonable request (last author).

## Abbreviations

BPD: Bronchopulmonary dysplasia
ELBW: Extremely low birth weight
ES: Enterostomy
ICH: Intracranial hemorrhage
LOS: Length of stay
NEC: Necrotizing enterocolitis
PA: Primary anastomosis
PDA: Patent ductus arteriosus
SIP: Spontaneous intestinal perforation
VLBW: Very low birth weight
